# α-Tocopheryloxyacetic acid: a novel chemotherapeutic that stimulates the antitumor immune response

**DOI:** 10.1186/bcr2808

**Published:** 2011-01-13

**Authors:** Tobias Hahn, Bhumasamudram Jagadish, Eugene A Mash, Kendra Garrison, Emmanuel T Akporiaye

**Affiliations:** 1 Robert W Franz Cancer Research Center, Earle A Chiles Research Institute, Providence Portland Medical Center, 4805 NE Glisan Street, Portland, OR 97213, USA; 2 Department of Chemistry and Biochemistry, University of Arizona, 1306 East University Boulevard, Tucson, AZ 85721, USA

## Abstract

**Introduction:**

α-Tocopheryloxyacetic acid (α-TEA) is a novel ether derivative of α-tocopherol that has generated interest as a chemotherapeutic agent because of its selective toxicity toward tumor cells and its ability to suppress tumor growth in various rodent and human xenograft models. We previously reported that oral α-TEA inhibited the growth of both a transplanted (4T1) and a spontaneous MMTV-PyMT mouse model of breast cancer.

**Methods:**

Because little is known about the possible immunological mechanisms underlying the *in vivo *α-TEA effects, we evaluated the impact of α-TEA therapy on the immune response by characterizing immune cell populations infiltrating the tumor site.

**Results:**

α-TEA treatment resulted in higher frequencies of activated T cells in the tumor microenvironment and twofold and sixfold higher ratios of CD4^+ ^and CD8^+ ^T cells to regulatory T cells, respectively. This finding was correlated with an increased ability of tumor-draining lymph node cells and splenocytes from α-TEA-treated mice to secrete interferon (IFN)-γ in response to CD3 or to mediate a cytolytic response in a tumor-specific fashion, respectively. That the α-TEA-mediated antitumor effect had a T cell-dependent component was demonstrated by the partial abrogation of tumor suppression when CD4^+ ^and CD8^+ ^T cells were depleted. We also determined the intratumoral cytokine and chemokine profile and found that α-TEA treatment increased intratumoral IFN-γ levels but decreased interleukin (IL)-4 levels, suggesting a shift toward a TH1 response. In addition, α-TEA induced higher levels of the inflammatory cytokine IL-6 and the chemokine CCL5.

**Conclusions:**

Taken together, these data suggest that α-TEA treatment, in addition to its direct cytotoxic effects, enhanced the anti-tumor immune response. This study provides a better understanding of the mechanisms of action of α-TEA and its effect on the immune system and may prove useful in designing immune-stimulating strategies to boost the antitumor effects of α-TEA in breast cancer patients.

## Introduction

Over the past several years, vitamin E α-tocopherol (α-TOH) analogs (VEA) have been evaluated for their antitumor activities. Of these analogs, α-tocopheryl succinate (α-TOS) and α-tocopheryloxyacetic acid (α-TEA) have been the most studied [1-9]. Both analogs have generated great interest as potential chemotherapeutic agents because they exhibit selective toxicity toward tumor cells [7,10-13] and suppress tumor growth in various rodent and human xenograft tumor models [5,7,9,11,14-18]. α-TEA structurally shares the phytyl tail and the chroman head with α-TOH, but differs from α-TOH in that the hydroxyl group at the number 6 carbon of the phenolic ring of the chroman head is replaced by an acetic acid residue that is attached by a nonhydrolyzable ether bond [7] which makes oral administration of α-TEA possible. In this respect, we reported that, when it is supplied to mice in their diet, α-TEA significantly inhibited the growth of a transplanted, highly metastatic breast cancer, dramatically reduced the incidence of lung metastases [9] and was able to delay the onset of and suppress tumor growth in a clinically relevant spontaneous MMTV-PyMT mouse model of breast cancer [18].

Recent data demonstrating that certain classes of chemotherapeutic drugs cause immunogenic tumor cell death, which leads to enhancement of antigen cross-presentation and stimulation of the antitumor immune response, have galvanized interest in chemotherapeutic agents as immune modulators [19-23]. It is well documented that one mechanism of VEA-mediated tumor cell death involves proapoptotic signaling and downregulation of survival pathways [2,24]. In addition, we have demonstrated by *in situ *analysis of tumor tissues in the MMTV-PyMT mouse spontaneous breast cancer model that apoptotic cell death is an important mechanism of α-TEA-mediated tumor suppression [18]. However, the majority of studies that have examined the mechanism of α-TOS- or α-TEA-induced anticancer activity have only focused on the proapoptotic nature of these analogs [3,24,25]. Therefore, little is known about the possible immunological mechanisms that underlie the *in vivo *antitumor effects of these VEAs. In this regard, we have shown that these VEAs synergize with *ex vivo *generated dendritic cells (DCs) to inhibit the growth of established primary mammary tumors and suppress the formation of spontaneously arising metastases [17,26,27]. This finding led us to hypothesize that the *in vivo *antitumor effects of α-TEA may have an immune component. In this report, we demonstrate that α-TEA increased the frequencies of activated CD4^+ ^and CD8^+ ^T cells in the tumor microenvironment, induced a tumor-specific cytotoxic lymphocyte response and resulted in higher CD4^+^-to-Treg and CD8^+^-to-Treg ratios, as well as that the α-TEA-mediated antitumor effect was dependent on the T cell response. α-TEA treatment also modulated the intratumoral cytokine and chemokine milieus. Most notably, α-TEA increased intratumoral interferon (IFN)-γ levels but decreased interleukin (IL)-4 levels, suggesting a shift toward a T cell-mediated T helper type 1 (TH1) response. This study demonstrates for the first time the immunostimulatory activities of α-TEA. This knowledge that α-TEA can stimulate the immune system while directly killing tumor cells may prove useful in designing combination immunotherapy strategies to boost the antitumor effects of α-TEA in breast cancer patients.

## Materials and methods

### Preparation of α-tocopheryloxyacetic acid

α-TEA, 2,5,7,8-tetramethyl-(2R-(4R,8R,12-trimethyltridecyl) chroman-6-yloxy) acetic acid, was synthesized at The Arizona Cancer Center Synthetic Shared Resource at The University of Arizona (Tucson, AZ) using modified previously described methods [7,28]. To a suspension of NaH (4.3 g, 181 mM) in dry tetrahydrofuran (THF, 400 mL) under argon at 0°C was added a solution of *(R,R,R)*-α-tocopherol (59.9 g, 139 mM) in dry THF (200 mL). The mixture was stirred at 0°C for 30 minutes, and ethyl bromoacetate (27.8 g, 167 mM, 18.5 mL) was added via syringe. The reaction mixture was stirred for 3.5 hours at 0°C and for 24 hours at room temperature (RT). The reaction mixture was poured slowly into ice water (500 mL) and extracted with ether (200 mL × 4). The ether extracts were combined, washed with brine, dried over MgSO_4_, filtered and evaporated under reduced pressure to produce the ethyl ester of α-TEA (71.0 g, 100%) as viscous oil. The ester was dissolved in THF (700 mL), 10% aqueous KOH solution (260 mL, 419 mmol) was added and the mixture was stirred at room temperature for 6 hours. The reaction was quenched with water (300 mL) and the pH of the solution adjusted to pH 3 using 1N HCl. The product was extracted with ether (200 mL × 4). The combined ether extracts were washed with brine, dried over MgSO_4_, filtered, and evaporated to give impure α-TEA as viscous yellow oil. Crystallization of the oil from pentane at -20°C produced pure α-TEA (55.0 g, 113 mM, 82%) as a white waxy solid, mp 54°C [7] (mp 54°C to 55°C), [α]_D_^23 ^+0.8 (*c *1, CHCl_3_). Purity and identity were confirmed by high-performance liquid chromatography and nuclear magnetic resonance analysis. ^1^H NMR (600 MHz, CDCl_3_) δ 0.85 (12, m), 1.02 to 1.14 (7, m), 1.16 to 1.43 (14, m), 1.49 to 1.55 (3, m), 1.74 to 1.82 (2, m), 2.07 (3, s), 2.13 (3, s), 2.17 (3, s), 2.56 (2, m), 4.36 (2, m); ^13^C NMR (150 MHz, CDCl_3_) δ 11.7, 11.8, 12.7, 19.6, 19.7, 20.6, 20.9, 22.6, 22.7, 23.8, 24.4, 24.7, 27.9, 31.1, 32.6, 32.7, 37.2, 37.3, 37.4, 39.3, 40.0, 69.1, 74.9, 117.7, 123.2, 125.4, 127.3, 147.0, 148.5, 173.3. HRMS (LCQ/FTICR, Thermo Fisher Scientific, Waltham, MA, USA) calculated for C_31_H_51_O_4 _487.3793 [M-H]^-^, observed 487.3794.

### Tumor cells and cell culture

The 4T1 tumor cell line is a variant of 410.4, a tumor subline that was isolated from a spontaneous mammary tumor in a BALB/cfC3H mouse. The 4T1 tumor is poorly immunogenic and highly metastatic and spontaneously metastasizes to the liver, lungs, bone marrow and brain [29-31]. The Renca cell line was derived from a spontaneous renal cortical adenocarcinoma in BALB/c mice [32]. The tumor cells (4T1 and Renca) were maintained in Dulbecco's modified Eagle's medium (DMEM; Lonza, Walkersville, MD, USA), containing 100 U/mL penicillin, 100 mg/mL streptomycin (HyClone Laboratories, Logan, UT, USA), 0.025 mg/mL amphotericin B (HyClone Laboratories) and 10% fetal bovine serum (FBS; Lonza).

### Animal studies

Six- to eight-week-old female BALB/c mice were purchased from Harlan Laboratories (Indianapolis, IN, USA) and housed at the animal facility of the Earle A. Chiles Research Institute in accordance with the Principles of Animal Care (National Institutes of Health publication 85-23). All studies were reviewed and approved by the institutional animal care and use committee of the Earle A. Chiles Research Institute. For the transplantable tumor model studies, 5 × 10^4 ^viable 4T1 breast cancer cells were injected subcutaneously into the right mammary fat pad of mice. The mice received a nutrient-matched control diet (Harlan Teklad, Madison, WI, USA) until tumor establishment (day 10 post-tumor implantation, average tumor size of ~15 mm^2^) and were then switched to mouse chow containing α-TEA. α-TEA was incorporated into the AIN93G diet by Harlan Teklad at a concentration of 3 g α-TEA/kg chow (0.3%), resulting in a dose of ~6 mg of α-TEA per day per mouse (equivalent to ~300 mg of α-TEA/1 kg body wt). For T cell depletions, mice were injected intraperitoneally with 200 μg of CD4-specific (GK1.5) and CD8-specific (2.43) antibodies (BioXCell, West Lebanon, NH, USA) on day 9 post-tumor injection (1 day before initiation of α-TEA therapy) and weekly thereafter. Control animals received rat immunoglobulin G 2b (IgG2b) (LTF-2) isotype control antibody (BioXCell). Depletion of T cells was monitored in peripheral blood by flow cytometry, and the frequency of CD4^+ ^and CD8^+ ^T cells was less than 0.5%. Tumor growth was monitored by measuring the tumor length (L) and width (W) using calipers and calculating the tumor area as A = (L × W).

### Isolation of lymph node, splenic and tumor infiltrating immune cells

Tumor-draining inguinal lymph nodes were resected and pushed through a 70-μm nylon sieve (BD Biosciences Discovery Labware, Two Oaks, CA, USA) to produce a single cell suspension. The cells were then washed (300 × *g *for 7 minutes) and filtered through a 40-μm nylon sieve (BD Biosciences). Spleens were resected and pushed through a 70-μm nylon sieve to produce a single cell suspension. After red blood cell lysis, the cells were washed (300 × *g *for 7 minutes) and filtered through a 40-μm nylon sieve. To isolate tumor-infiltrating immune cells (TICs), tumors were resected and minced using a scalpel blade in a triple enzyme digestion mix containing 10 mg/mL collagenase type IV (Worthington Biochemical Corp., Lakewood, NJ, USA), 1 mg/mL hyaluronidase (Sigma-Aldrich, St. Louis, MO, USA), 200 μg/mL DNAse I (Roche Applied Sciences, Indianapolis, IN, USA) in Hanks' Balanced Salt Solution (Lonza). The tumors were then incubated with agitation (37°C for 45 minutes). After the addition of 10 mM ethylenediaminetetraacetic acid, the digestion product was incubated for another 15 minutes. Subsequently, the digested tissue was pushed sequentially through 70-μm and 40-μm nylon sieves, washed (300 × *g *for 7 minutes), overlaid on Ficoll (FicoLite-LM; Atlanta Biologicals, Lawrenceville, GA, USA) and centrifuged (1,500 × *g *for 25 minutes without brake). The interface was collected and washed twice (300 × *g *for 7 minutes).

### *In vitro *stimulation of lymph node cells

Inguinal tumor-draining lymph nodes (TDLN) were resected, and a single cell suspension was prepared as described above. Cells were pooled from three mice per treatment group, and 1 × 10^6 ^TDLN cells per well were incubated for 48 hours in a 24-well tissue culture plate in 1 mL of Roswell Park Memorial Institute 1640 (RPMI 1640) medium containing 100 U/mL penicillin, 100 mg/mL streptomycin (HyClone Laboratories), 0.025 mg/mL amphotericin B (HyClone Laboratories), 70 μM β-2-mercaptoethanol (Sigma-Aldrich), 2 mM L-glutamine (Lonza), 1 mM sodium pyruvate (Lonza), 1× nonessential amino acids (Lonza), 10 mM HEPES (4-(2-hydroxyethyl)-1-piperazineethanesulfonic acid; Lonza) and 10% FBS (Lonza). For CD3 stimulation, wells were coated with 5 μg/mL anti-CD3 antibody (BD Pharmingen, San Jose, CA, USA) for 24 hours at 4°C and washed with phosphate-buffered saline before addition of lymph node cells. Supernatants were collected after 48 hours and stored frozen (-80°C) until analysis. IFN-γ levels were determined by enzyme-linked immunosorbent assay (ELISA; eBioscience, San Diego, CA, USA, and BD Biosciences) according to the manufacturers' instructions.

### Cytotoxicity assay

Splenocytes were isolated as described above and pooled from three mice per treatment group (2 × 10^6 ^cells/well in 2 mL RPMI 1640) and cultured in 24-well tissue culture plates in the presence of 100 μg/mL 4T1 tumor cell freeze-thaw lysate and 10 U/mL IL-2 for 6 days. A standard ^51^Cr release cytotoxicity assay was performed [33] using Renca cells as irrelevant targets. Briefly, cultured splenocytes (effectors) were diluted to achieve a range of effector-to-target ratios and added in triplicate to ^51^Cr-labeled target cells (4T1 or Renca) in U-bottomed 96-well plates. After incubation for 6 hours (37°C, 5% CO_2_), 25 μL of supernatant was added to 150 μL of Optiphase Supermax scintillation fluid (Perkin-Elmer, Waltham, MA, USA) and mixed by shaking for 10 minutes. The released ^51^Cr (experimental) was quantified using a Trilux 1450 MicroBeta Liquid Scintillation Counter (Perkin-Elmer). The total ^51^Cr amount incorporated into tumor cells was determined by lysing ^51^Cr-labeled cells with Triton X-100 (Sigma-Aldrich). Spontaneous ^51^Cr release was measured by using supernatant from ^51^Cr-labeled cells that were incubated with media alone. The percentage of specific tumor cell lysis was calculated using the following formula: % cytotoxicity = [(Experimental) - (Spontaneous)]/[(Total) - (spontaneous)] × 100. Spontaneous release did not exceed 15% and 25% for 4T1 and Renca cells, respectively.

### Flow cytometric analysis

Lymph node cells and TICs were stained with fluorophore-conjugated antibodies and analyzed by five- or six-color flow cytometry on an LSR-II flow cytometer (BD Biosciences). Antibodies used for five-color flow cytometry were CD3-FITC (eBioscience), CD4-APC-H7 (BD Pharmingen), CD8-Pacific Orange (Caltag, Carlsbad, CA, USA), CD25-PE (Caltag) and Foxp3-APC (eBioscience). For intracellular Foxp3 staining, cells were permeabilized and fixed using a fixation and permeabilization kit (eBioscience). Antibodies used for six-color flow cytometry were CD3-PerCP-Cy5.5 (eBioscience), CD4-APC-H7 (BD Pharmingen), CD8-Pacific Orange (Caltag), CD25-APC (eBioscience), CD44-FITC (eBioscience) and CD62L-PE-TR (Caltag). Cell viability was assessed using the LIVE/DEAD Fixable Violet stain (Invitrogen, Carlsbad, CA, USA). Data were acquired using DIVA software (BD Biosciences) and analyzed using either FlowJo v8.8.4 (Tree Star Inc., Ashland, OR, USA) or Winlist™ 7.0 software (Verity House Software, Topsham, ME, USA), including combination function (FCOM) analysis.

### Multiplex analysis

To identify the soluble factors in the tumor microenvironment, tumors were resected and 100 mg of tumor tissue was minced using scissors in 500-μL Bio-Plex tissue lysis buffer (Bio-Rad, Hercules, CA, USA) containing Complete Lysis-M protease inhibitor cocktail (Roche Applied Sciences). Subsequently, the tumor tissue was further homogenized using a rotor stator homogenizer (PRO Scientific Inc., Oxford, CT, USA). The lysate was clarified (10,000 × *g *for 30 minutes at 4°C), and the supernatant was stored at -80°C until analysis. The samples were thawed on ice, and protein content was determined by bicinchoninic acid protein assay (Thermo Scientific, Rockford, IL, USA). Cytokines and chemokines were assayed using multiplex luminescent beads (Bio-Plex Pro Cytokine custom assay; Bio-Rad) according to the manufacturer's instructions and analyzed using a Bio-Plex Analyzer (Bio-Rad). Fluorescence intensity was transformed into cytokine concentrations using the Bio-Plex manager 5.0 software (Bio-Rad) and normalized to protein content of the sample. The following cytokines and chemokines were evaluated: IL-1β, IL-2, IL-4, IL-5, IL-6, IL-10, IL-12p70, IL-13, IL-17, granulocyte macrophage colony-stimulating factor (GM-CSF), IFN-γ, chemokine C-C motif ligand 2 (CCL2), chemokine C-C motif ligand 3 (CCL3), chemokine C-C motif ligand 4 (CCL4), chemokine C-C motif ligand 5 (CCL5) and tumor necrosis factor-α.

### Statistical analysis

The statistical significance of differences among data sets of treatment groups was assessed by using Student's *t*-test for pairwise comparisons or for comparisons of multiple groups by one-way analysis of variance (ANOVA) with Tukey's Honestly Significant Difference test to adjust for multiple comparisons. To compare tumor growth rates, growth curves were transformed to linearity and linear regression analysis was used to determine slopes that were then compared by *t*-test. Survival curves were estimated using the method of Kaplan and Meier, and log-rank tests were performed to assess differences in the hazard rates. All analyses were performed using Prism software (GraphPad, San Diego, CA, USA). *P *values ≤0.05 were considered indicative of significant differences between data sets.

## Results

### Oral α-TEA therapy inhibits the growth of established breast tumors and prolongs survival

**Figure 1:**
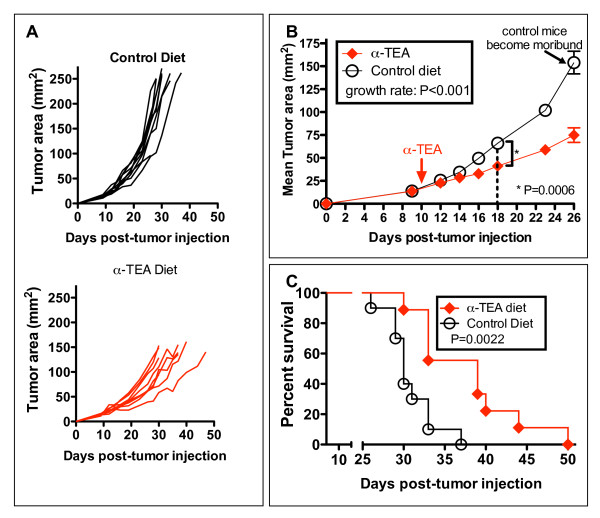
**Oral α-tocopheryloxyacetic acid (α-TEA) decreases breast tumor growth and prolongs survival**. BALB/c mice with established 4T1 mammary tumors (day 10, ~15 mm^2^) received oral α-TEA in their diet (~6 mg/day, equivalent to 300 mg/kg body wt) or a nutrient-matched control diet. **(A) **Individual tumor areas. **(B) **Mean tumor areas ± SEM (*n *= 10 mice per group). Difference in average tumor area first reaches significance on day 18 posttumor area (dotted line). **(C) **Kaplan-Meier analysis of survival.

We and others have previously demonstrated that α-TEA is effective at inhibiting the tumor growth of various rodent and human xenograft tumor models [5,7,9,11,14-18]. Our research has focused on the effects of α-TEA on breast cancer using the transplantable 4T1 murine breast cancer tumor model. Using a stringent experimental design of treating tumors that had been established for 10 days and were clearly palpable (~15 mm^2^), we showed that α-TEA (300 mg/kg body wt) was able to significantly suppress tumor growth (Figures 1A and 1B). This reduction in tumor size became apparent after ~8 days of α-TEA treatment (day 18 post-tumor cell injection) (Figure 1B) and resulted in a 1.6-fold reduction in average tumor area compared with mice on a control diet (*P *= 0.0006). On day 26 post-tumor injection, immediately before untreated animals started to become moribund (that is, when the tumor area reached ≥150 mm^2 ^or the mice died due to metastatic burden), α-TEA treatment resulted in a 2.1-fold reduction in average tumor size compared with untreated mice (*P *< 0.0001). Furthermore, α-TEA significantly prolonged the median survival by 9 days (Figure 1C) compared with the control diet group.

### α-TEA increases the frequency of activated T cells and promotes a less immune-suppressive environment in tumors

**Figure 2:**
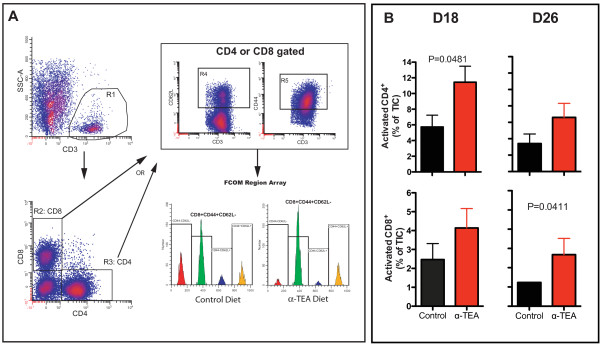
**Effect of α-TEA on activation status of tumor-infiltrating T cells**. BALB/c mice with established 4T1 mammary tumors (day 10) received oral α-TEA in their diet. On day 18 (D18) and day 26 (D26) posttumor injection, tumor-infiltrating immune cells (TICs) were isolated and analyzed by polychromatic flow cytometry. **(A) **After applying a live cell gate, CD3^+ ^cells were identified by the R1 gate. Subsequently, CD8^+ ^and CD4^+ ^T cells were delineated with the R2 and R3 gates and were then further interrogated for CD62L (R4) and CD44 (R5) expression. On the basis of the defined positive staining regions, combination function analysis (FCOM) calculated four phenotypes (FCOM region array). Activated T cells were identified as CD4^+^CD44^+^CD62L^- ^or CD8^+^CD44^+^CD62L^-^. Representative FCOM arrays from CD8^+ ^T cells are shown. **(B) **TICs were analyzed from three individual mice in each of two independent experiments. Subphenotype frequencies are based on all live tumor-infiltrating cells.

Although chemotherapy is commonly thought of as immunosuppressive, recently it has become apparent that certain classes of chemotherapeutic drugs cause immunogenic tumor cell death and may act as immune modulators [19-23]. Our own studies on the tumor-selective VEAs α-TOS and α-TEA have shown that when they are administered in combination with *ex vivo *generated DCs for the treatment of established mammary cancers, they synergize with DCs to inhibit tumor growth [17,26,27]. This finding led us to hypothesize that the antitumor effect of α-TEA may have an immune component. Therefore, we examined the frequency and activation status of T cells infiltrating the tumor site in mice with established 4T1 tumors (Figures 2A and 2B). We characterized the immune cell composition at day 18 post-tumor implantation when α-TEA tumor suppression first became apparent (Figure 1B). In addition, we analyzed the activation status of T cells immediately before mice on the control diet became moribund because of tumor burden (day 26 post-tumor injection). Activated T cells were identified by polychromatic flow cytometry as CD4^+^CD44^+^CD62L^- ^or CD8^+^CD44^+^CD62L^- ^cells after gating on live, CD3-expressing cells (Figure 2A). Phenotypic characterization of TICs revealed that α-TEA caused a significant increase (*P *= 0.0481) in the frequency of activated CD4^+ ^T cells in the tumor microenvironment on day 18 post-tumor injection (Figure 2B). The frequency of activated CD4^+ ^T cells trended higher on day 26 post-tumor injection. In addition, α-TEA caused a moderate increase of the average frequency of activated tumor-infiltrating CD8^+ ^T cells on day 18, which reached statistical significance (*P *= 0.041) on day 26.

**Figure 3:**
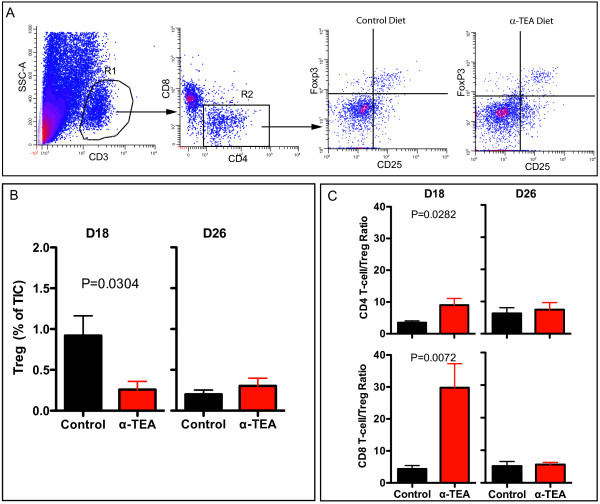
**Effect of α-TEA on regulatory T cells (Treg) in the tumor microenvironment**. BALB/c mice with established 4T1 mammary tumors (day 10) received oral α-TEA in their diet (~6 mg/day). On day 18 (D18) and day 26 (D26) post-tumor injection, TICs were isolated and analyzed by polychromatic flow cytometry. **(A) **After applying a live cell gate, CD3^+ ^cells were delineated by the R1 gate. Subsequently, CD4^+ ^T cells (R2) were further interrogated for CD25 and Foxp3 expression. Tregs were identified as CD3^+^CD4^+^CD25^+^Foxp3^+ ^cells. Representative flow cytometric analyses for D18 are shown. **(B) **Treg frequency. **(C) **Ratio of Treg to either non-Treg CD4^+ ^(CD4^+^Foxp3^-^) or CD8^+ ^T cells. TICs were analyzed from three individual mice from each of two independent experiments. Subphenotype frequencies are based on all live tumor-infiltrating cells.

The increase in the frequency of activated T cells was associated with a reduction in the frequency of regulatory T cells (Treg). On day 18, when the tumor growth curves started to diverge, α-TEA caused a 3.5-fold reduction in the frequency of Treg cells in the tumor microenvironment in comparison to mice on the control diet (Figure 3B). On day 26, the Treg frequency in the control mice was comparable to that of α-TEA-treated mice.

When we evaluated the ratio of effector T cells to Tregs (Figure 3C) in mice on the control diet, we found that at day 18 the ratios of (1) non-Treg CD4^+ ^T cells (CD4^+^/FoxP3^-^) to Treg cells (CD4^+^/CD25^+^/FoxP3^+^) and (2) CD8^+ ^T cells to Treg cells were both ~4. This was in contrast to α-TEA-treated animals, in which these ratios were significantly higher: ~10 (*P *= 0.0204) for non-Treg CD4^+ ^T cells and ~30 (*P *= 0.0072) for CD8^+ ^T cells. However, at day 26, there was no difference in the average T cell-to-Treg ratio between the two treatment groups.

### α-TEA treatment increases responsiveness of lymph node cells and induces a tumor-specific immune response

**Figure 4:**
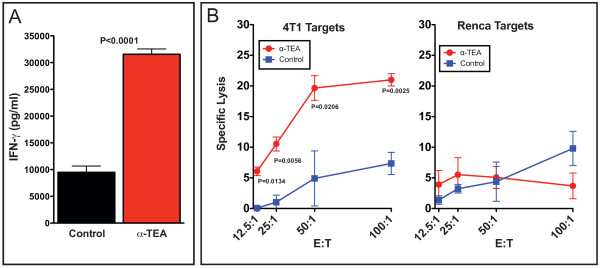
**α-TEA results in increased responsiveness to T cell receptor (TCR) stimulation and induced a tumor-specific cytotoxic response**. BALB/c mice with established 4T1 mammary tumors (day 10) received oral α-TEA in their diet for 16 days (day 26 post-tumor injection). **(A) **Tumor-draining lymph node cells were isolated, pooled from three mice per group and restimulated *in vitro *with plate-bound anti-CD3 antibody for 48 hours. Interferon (IFN)-γ secretion was determined by enzyme-linked immunosorbent assay. Combined results from two independent experiments are shown. **(B) **Splenocytes were isolated from three mice per group, pooled and restimulated *in vitro *for 6 days with 4T1 tumor cell lysate and then incubated with ^51^Cr-labeled 4T1 or Renca cells for 6 hours. ^51^Cr release into the supernatant was measured.

Next we wanted to determine whether α-TEA treatment influenced the responsiveness of T cells to T cell receptor (TCR) stimulation and induced a cytotoxic T cell response. For this purpose, tumor-draining lymph node (TDLN) cells were isolated on day 26 post-tumor injection (after 16 days of α-TEA treatment) and stimulated *in vitro *with an agonistic CD3-specific antibody to determine IFN-γ secretion as a surrogate marker for T cell activation. Our results show (Figure 4A) that the TDLN cells isolated from the α-TEA-treated mice responded with a threefold higher (*P *< 0.0001) secretion of IFN-γ in comparison to the TDLN cells from mice on the control diet. To determine whether α-TEA also induced a tumor-specific response, we assessed the ability of T cells isolated from α-TEA-treated mice to cause lysis of 4T1 tumor cells. Splenocytes were restimulated *in vitro *with 4T1 tumor cell lysate, and tumor-specific cytolytic activity was determined by an *in vitro *^51^Cr release assay using 4T1 and Renca cells as targets. The data in Figure 4B show that α-TEA treatment significantly increased the lysis of 4T1 target cells approximately fivefold compared to no treatment. This increased cytolytic activity by splenocytes from α-TEA-treated mice was specific to 4T1 tumor cells, as syngeneic, irrelevant Renca targets were minimally lysed at levels similar to the cytolytic ability of splenocytes from mice on the control diet (Figure 4B).

### The antitumor effect of oral α-TEA has a T cell-dependent component

**Figure 5:**
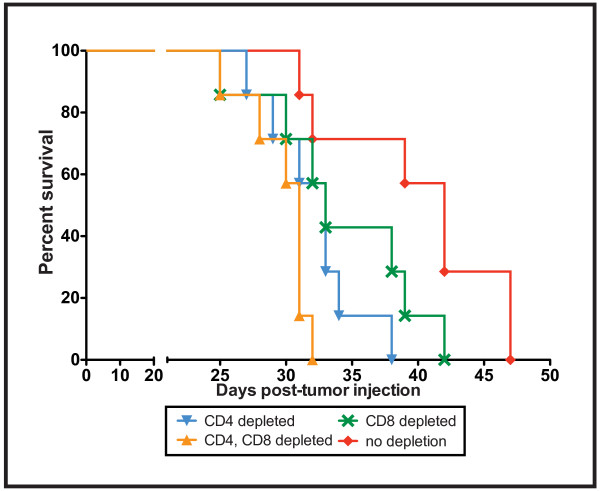
**α-TEA-mediated tumor suppression has a T cell-dependent component**. BALB/c mice with established 4T1 mammary tumors were depleted of T cells by intraperitoneal injection of CD4- and/or CD8-specific antibodies and received α-TEA in their diet (~6 mg/day, equivalent to 300 mg/kg body wt). Immunocompetent animals (not depleted) received isotype antibody and α-TEA. Kaplan-Meier analysis of survival is shown (*n *= 7 mice per group).

Having shown the recruitment and activation of T cells within tumors of α-TEA-treated mice, we wanted to determine whether T cells play a nonredundant role in α-TEA-mediated antitumor activity. To accomplish this, tumor-bearing mice on the α-TEA diet were depleted of CD4^+ ^and/or CD8^+ ^T cells and assessed for survival (Figure 5). The median survival of immune-sufficient mice treated with α-TEA was 42 days, in contrast to α-TEA-treated mice lacking both CD4^+ ^and CD8^+ ^T cells, which survived only until day 31 (*P *= 0.0021). Depletion of either CD4^+ ^or CD8^+ ^T cells in α-TEA-treated mice only moderately extended the median survival to 33 days. The data suggest that both CD4^+ ^and CD8^+ ^T cells play nonredundant roles in α-TEA-mediated antitumor activity.

### α-TEA treatment modulates the cytokine and chemokine milieus in the tumor microenvironment

Cytokines and chemokines play an important role in attracting or inhibiting the infiltration of immune cells into the tumor microenvironment and thus contribute to tumor growth or rejection. Therefore, we analyzed the cytokine and chemokine profiles of the microenvironment of α-TEA-treated tumors on day 21 post-tumor injection. The results in Table 1 show that α-TEA treatment induced a more proinflammatory cytokine and chemokine environment as evidenced by 2.4-fold and 2.3-fold higher levels of IL-6 and CCL5 (RANTES), respectively, compared to tumors from animals on the control diet. The levels of the proinflammatory cytokine IL-1β and the proinflammatory chemokines CCL2 (MCP-1), CCL3 (MIP1-α) and CCL4 (MIP1-α) trended higher in the α-TEA-treated tumors. α-TEA treatment also resulted in a 2.2-fold increase of IFN-γ level and a 2.7-fold decrease of IL-4 level, suggesting a shift toward a TH1-mediated antitumor immune response. Interestingly, α-TEA therapy also resulted in a 2.8-fold increase of IL-5 in the tumor.

**Table 1:** Cytokine and chemokine profiles in the tumor microenvironment

Cytokine/chemokine	Control diet (pg/mg protein ± SEM)	*n*	α-TEA diet (pg/mg protein ± SEM)	*n*	*P *value
IL-1β	306.7 ± 75.2	7	463.4 ± 133.5	7	0.3177
IL-2	3.2 ± 0.3	3	6.2 ± 1.4	3	0.0989
IL-4	3.7 ± 1.6	7	0.6 ± 0.2	6	0.0044
IL-5	3.8 ± 0.4	7	10.7 ± 4.7	7	0.0379
IL-6	8.3 ± 1.8	7	19.8 ± 2.6	7	0.0016
IL-10	4.1 ± 0.6	3	9.5 ± 2.7	3	0.1143
IL-12p70	ND	7	ND	7	-
IL-13	32.9 ± 2.9	3	35.9 ± 9.9	3	0.9472
IL-17	3.2 ± 0.4	7	3.9 ± 0.4	7	0.4557
GM-CSF	22.9 ± 4.7	7	31.3 ± 4.4	7	0.1745
IFN-γ	5.9 ± 0.5	3	12.9 ± 2.8	3	0.0478
CCL2 (MCP-1)	1,357.2 ± 411.3	7	2,712.8 ± 679.9	7	0.1055
CCL3 (MIP1-α)	324.7 ± 49.9	3	837.5 ± 256.2	3	0.0514
CCL4 (MIP-1β)	36.5 ± 3.7	7	61.1 ± 12.5	7	0.1440
CCL5 (RANTES)	193.6 ± 42.6	7	447.2 ± 109.5	7	0.0379
TNF-α	12.1 ± 1.3	7	13.3 ± 0.8	7	0.4415

BALB/c mice with established 4T1 mammary tumors (day 10) received oral α-TEA in their diet. On day 21 posttumor injection, tumors (*n *= 3 to 7 per group) were resected and equal amounts of tissue were homogenized in tissue lysis buffer containing protease inhibitors. The lysates were clarified by centrifugation, and cytokine and chemokine levels were determined using multiplex luminescent beads. Levels (means ± SEM) were normalized by protein content of the lysate.

α-TEA, α-tocopheryloxyacetic acid; CCL2 (MCP-1), chemokine C-C motif ligand 2 (monocyte chemotactic protein-1); CCL3 (MIP1-α), chemokine C-C motif ligand 3 (macrophage inflammatory protein 1-α); CCL4 (MIP-1β), chemokine C-C motif ligand 4 (macrophage inflammatory protein 1β); CCL5 (RANTES), chemokine C-C motif ligand 5 (regulated upon activation, normal T-cell expressed, and secreted); GM-CSF, granulocyte macrophage colony-stimulating factor; IFN-γ, interferon-γ; IL, interleukin;  ND, not detectable; TNF-α, tumor necrosis factor-α.

## Discussion

In this study, we determined whether there is an immunological basis for α-TEA-mediated antitumor activity [7,9,18] in addition to the previously described proapoptotic and mitochondria-destabilizing effects of the VEAs on tumor cells [3,24,25,34]. To examine whether α-TEA treatment modulated a T cell-mediated antitumor immune response, we evaluated the activation status of tumor-infiltrating T cells. While systemic antitumor immune responses in the periphery may be informative, it is the quality of the local immune responses at the tumor site itself that are most important, as they determine tumor progression or rejection. The phenotypic characterization of TICs revealed that α-TEA caused an increase in the frequency of activated CD4^+ ^T cells in the tumor microenvironment on day 18 post-tumor cell injection. This trend was still present on day 26 post-tumor cell injection. In addition, compared with mice on the control diet, the frequency of activated tumor-infiltrating CD8^+ ^T cells was significantly higher on day 26 in the α-TEA-treated mice. The finding of higher proportions of T cells with an activated phenotype in the tumor suggests that α-TEA-treated mice were able to mount a more potent antitumor immune response that contributed to tumor suppression. This notion was supported by the finding of threefold higher IFN-γ secretion by TDLN cells after TCR stimulation and, more important, a fivefold increase of tumor-specific cytolytic activity by splenocytes from α-TEA-treated animals compared with untreated animals.

To establish the involvement of the immune system in α-TEA-mediated antitumor response, α-TEA-treated, tumor-bearing mice were depleted of CD4^+ ^and CD8^+ ^T cells and evaluated for survival. Our results show that the T cell response significantly contributed to the α-TEA-mediated prolongation of survival. The necessity of both T cell subsets for the α-TEA antitumor effect also correlated with the finding of higher frequencies of activated CD4^+ ^and CD8^+ ^T cells in the tumor microenvironment.

It is well documented that the presence of immune cells within tumors does not necessarily predict a favorable clinical outcome in cancer patients. In the majority of cancers, tumor progression occurs even in the presence of immune cells, such as cytotoxic CD8^+ ^T cells and natural killer cells [35], that are frequently associated with cytotoxic effector functions. As Tregs constitute an important component of the host cells attracted to the tumor site, the presence of this immune suppressive cell population may contribute to blunting an effective antitumor immune response and thus increase tumor progression [36-38]. An increased frequency of Tregs in tumors, peripheral blood and secondary lymphoid organs is a common occurrence in animal tumor models [38-40] and cancer patients [37,41-43], and it is associated with metastatic spread and poor survival [36-38]. Determination of the frequency of Tregs at day 18, when the tumor growth curves started to diverge, showed that α-TEA treatment resulted in a lower frequency of Tregs in the tumor microenvironment. However, at day 26 post-tumor cell injection, the Treg frequency in the untreated mice had decreased to levels similar to the Treg frequency in the α-TEA-treated mice. The decreased frequency of Tregs in the untreated mice could be due to the larger tumor size in comparison to the α-TEA-treated animals. Furthermore, the ratios of CD4^+ ^or CD8^+ ^T cells to Treg was twofold and sixfold higher, respectively, in the tumors of the α-TEA-treated animals at day 18 compared with the control mice, and these data corroborate previous reports in which higher CD8^+ ^cytotoxic T cell-to-Treg ratios in the tumor or in lymphoid organs were associated with improved prognosis [44-46]. These findings suggest that α-TEA treatment created a less suppressive tumor microenvironment that allowed effector T cells to better eliminate tumor cells.

The notion that α-TEA treatment shaped a tumor environment that is more favorable for immune rejection is also supported by alterations in the cytokine and chemokine profiles in the tumor milieu of α-TEA-treated mice. In particular, α-TEA treatment resulted in a TH1-to-TH2 ratio (IFN-γ-to-IL-4 ratio) of ~22 for the α-TEA-treated group in comparison to ~2 for the control group. This higher TH1-to-TH2 ratio suggests a more robust effector T cell response, which was correlated with higher frequencies of activated CD4^+ ^and CD8^+ ^T cells and higher CD4^+ ^T cell-to-Treg and CD8^+ ^T cell-to-Treg ratios in the tumors of the α-TEA-treated mice. Although α-TEA also increased levels of IL-5, generally considered to be a TH2-associated cytokine, IL-5 may attract TH2-dependent antitumor effectors such as eosinophils [47,48]. Furthermore, α-TEA treatment modulated the tumor microenvironment in a proinflammatory fashion, as we found significantly higher IL-6 and CCL5 levels and a trend toward higher levels of other proinflammatory mediators (IL-1β, CCL2, CCL3, CCL4). IL-6 is a pleiotropic cytokine affecting multiple immune cell subsets. Notably, it has been shown to antagonize the immunosuppressive effects of TGF-β in the tumor microenvironment [49]. Similarly to human breast cancer [50,51], the 4T1 tumor model [52] produces large quantities of TGF-β that suppress an effective antitumor immune response [53,54]. Previously, it was shown that 4T1 cells produce various chemokines, including CCL2, CCL3, CCL4 and CCL5 [55], that we also detected in the tumor microenvironment, with CCL5 levels being significantly higher in tumors in α-TEA-treated mice. The higher intratumoral CCL5 levels after α-TEA treatment may contribute to the attraction of T cells to the tumor bed, as has been shown for inflammatory sites, including tumor sites [56,57]. However, it is not clear whether the 4T1 cells are the only source of chemokines, since tumor-infiltrating host cells also secrete these chemokines. Taken together, the cytokine and chemokine profiles suggest that α-TEA treatment promotes a proinflammatory environment that is more conducive to tumor suppression.

The ability of α-TEA to modulate the immune response corroborates studies with other cytoreductive chemotherapeutic agents, such as the taxanes [58] and anthracyclines [19]. The taxane docetaxel has been demonstrated not only to enhance the antitumor cytotoxic response but also to modulate the tumor microenvironment by decreasing the infiltration of myeloid-derived suppressor cells [59]. Using the EL4 tumor model, Maccubbin *et al*. [60] also showed that the anthracycline doxorubicin enhanced the cytotoxic T lymphocyte response, but it was more recently revealed that the mechanism of this immune modulation includes the translocation of calreticulin to the surface of tumor cells alerting DCs to take up dying tumor cells [20], the induction of heat shock proteins (Hsps) [61] and the secretion of high-mobility group 1 proteins [62]. We hypothesize that α-TEA may cause immunogenic tumor cell death, resulting in the upregulation and/or release of endogenous damage-associated molecular pattern molecules, such as Hsps [62-70] and the release of putative tumor-associated antigens that can be ingested by DCs and efficiently cross-presented to stimulate naïve tumor-specific T lymphocytes [71,72]. Our previous results showing that α-TOS- or α-TEA-treated tumor cells upregulated and translocated Hsps to the cell surface [17,27] and that supernatant from α-TOS- or α-TEA-treated tumor cells stimulated DC activation and maturation [17,27] lend support to this notion. Although our findings clearly demonstrate that α-TEA enhances the antitumor immune response by coopting the adaptive T cell response, the contribution of the direct killing of tumor cells by α-TEA cannot be overlooked. We previously reported that α-TEA induces apoptotic death of 4T1 tumor cells *in vitro *[9] and confirmed that apoptosis is also involved in tumor reduction by α-TEA *in vivo *[18]. Furthermore, we have shown that α-TEA synergizes with DC vaccination to decrease the frequency of spontaneous tumors in the spontaneous MMTV-PyMT mouse breast cancer model [73], demonstrating the ability of α-TEA to act as an adjuvant.

## Conclusions

α-TEA increased the frequencies of activated CD4^+ ^and CD8^+ ^T cells, resulted in higher CD4^+^-to-Treg and CD8^+^-to-Treg ratios in the tumor microenvironment and induced tumor-specific cytotoxicity. In addition, α-TEA treatment modulated the intratumoral cytokine and chemokine milieus in a manner that suggests a shift toward a T cell-mediated TH1 response. To the best of our knowledge, this study demonstrates for the first time that, in addition to the direct cytotoxic effect on tumor cells, α-TEA suppresses *in vivo *breast tumor growth in a T cell-dependent manner.

## Abbreviations

ANOVA: one-way analysis of variance; α-TEA: α-tocopheryloxyacetic acid; α-TOH: α-tocopherol; α-TOS: α-tocopheryl succinate; DC: dendritic cell, DMEM: Dulbecco's modified Eagle's medium; HCl: hydrochloric acid; KOH: potassium hydroxide; MgSO_4_: magnesium sulfate; NK: natural killer cell; RPMI 1640: Roswell Park Memorial Institute 1640 medium; TCR: T cell receptor; TDLN: tumor-draining lymph nodes; TIC: tumor-infiltrating immune cell; TH1: T helper type 1; TH2: T helper type 2; THF: tetrahydrofuran; Treg: regulatory T cell; VEA: vitamin E analog.

## Competing interests

The authors declare that they have no competing interests.

## Authors' contributions

TH participated in the design of the study and performed the *in vivo *experiments, the characterization of the tumor-infiltrating immune cell populations and the cytokine and chemokine milieu of the tumor microenvironment, and drafted the manuscript. KG participated in the isolation of tumor-infiltrating immune cells. ETA conceived of the study, participated in its design, and helped to draft the manuscript. EAM and BJ synthesized the α-tocopheryloxyacetic acid. All authors read and approved the final manuscript.
